# Listening to the Whispers in Neuroimmune Crosstalk: A Comprehensive Workflow to Investigate Neurotrophin Receptor p75NTR Under Endogenous, Low Abundance Conditions

**DOI:** 10.3389/fimmu.2021.648283

**Published:** 2021-04-16

**Authors:** Benjamin W. Dorschner, Ralf Wiedemuth, Ann-Christin Funke, Marc Gentzel, Mary-Louise Rogers, Sebastian Brenner, Sebastian Thieme

**Affiliations:** ^1^ Experimental Hematology, Department of Pediatrics, University Clinic Carl Gustav Carus, Dresden, Germany; ^2^ Molecular Analysis - Mass Spectrometry, Center for Molecular and Cellular Bioengineering (CMCB), Technische Universitaet Dresden, Dresden, Germany; ^3^ Centre for Neuroscience, College of Medicine and Public Health, Flinders University, Adelaide, SA, Australia

**Keywords:** p75NTR, CD271 (p75NTR), mass spectrometry, endogenous immunoprecipitation, protein-protein interaction, PMDC05, A375 (human melanoma) cell line, neuroimmune crosstalk

## Abstract

Inflammatory conditions are critically influenced by neuroimmune crosstalk. Cytokines and neurotrophic factors shape the responses of both nervous and immune systems. Although much progress has been made, most findings to date are based on expression of recombinant (tagged) proteins. The examination of receptor interactions by immunoprecipitation (IP) at endogenous levels provides further insight into the more subtle regulations of immune responses. Here, we present a comprehensive workflow and an optimized IP protocol that provide step-by-step instructions to investigate neurotrophin receptor p75NTR at endogenous, low abundance levels: from lysate preparation and confirmation of receptor expression to antibody validation and successful detection of protein-protein interactions. We employ human melanoma cell line A375 to validate specific antibodies and IP conditions, and apply these methods to explore p75NTR interactions in human leukemic plasmacytoid dendritic cell line PMDC05 detecting 14-3-3ϵ:p75NTR interaction in this cell type. With p75NTR as an exemplary protein, our approach provides a strategy to detect specific interaction partners even under endogenous, low abundance expression conditions.

## Introduction

Despite the apparent differences between the nervous and immune systems, they share an intriguing similarity: both employ a system of cytokines and neurotrophic factors enabling them to transmit information from one system to the other (1). This neuroimmune crosstalk has been implicated in a wide range of reactions and conditions, e.g. neuroinfectious and autoimmune reactions, as well as allergic diseases (2–4). A special role has been proposed for neurotrophins, including Nerve Growth Factor (NGF) and their receptors (5, 6). p75NTR is a universal neurotrophin receptor that relays extracellular NGF signals to intracellular compartments, either as a single receptor, or in complex with other receptors (7). The aim of the current study was to establish a comprehensive workflow to investigate protein interactions of p75NTR in cell types with low endogenous expression. Given that to date most results stem from recombinant expression models, our approach may provide further insight into more subtle regulations of immune responses.

A prominent role in neuroimmune crosstalk has been proposed for neurotrophin NGF and p75NTR, a universal neurotrophin receptor with a single transmembrane domain (6, 8). Originally purified in the late 1970s and early 1980s from rodent sympathetic ganglia and human melanoma cells (9), p75NTR is expressed in central nervous system cells as well as a variety of immune cells (6, 8). Under inflammatory conditions, both NGF secretion and p75NTR expression are dynamically regulated (6, 8). These dynamic changes shape different immune responses depending on p75NTR expression levels [for review see (10)].

P75NTR is also expressed in conventional and plasmacytoid dendritic cells (pDC) (11, 12). Dendritic cells play a pivotal role in bridging innate and adaptive immune responses (13). Their capacity to present antigens to T lymphocytes is a key element in the initiation of effective immune responses (14). Conventional and plasmacytoid dendritic cell functionality is influenced by NGF through p75NTR (11, 12). Recently, we have shown that NGF binding to p75NTR on pDC modulates T cell priming, aggravating lung inflammation in a T_H_2-prone asthma model and alleviating disease progression in a T_H_1-prone diabetes model (12). Despite these effects, p75NTR expression levels show a considerable inter-individual variation in human pDC (12). Therefore, when investigating p75NTR and its interacting proteins, a method should be used that encompasses the entire dynamic range of endogenous expression from low to high abundance levels.

A standard method to study protein-protein interactions is immunoprecipitation (IP). Its principle is to capture a target protein (and interacting proteins) by use of a specific antibody coupled to a stationary matrix [for review see (15)]. Extensive research advanced the field extraordinarily and recent developments include e.g. single cell IP, multiplexed IP, and time-resolved analysis of protein-protein interaction networks (16–18). In general, many variables determine the success of IP experiments, e.g. matrix material and antibody coupling, lysis and washing buffers as well as elution conditions. The investigation of proteins at endogenous expression levels is further limited by factors such as relative abundance and the quality of antibodies used to detect the protein in question. Many experiments rely on the recombinant expression of (tagged) proteins, thereby overcoming these limitations. Interestingly, there is an increasing body of evidence that expression levels of receptor proteins may regulate the function of their downstream mediators [e.g. (19), for review see (10)]. This emphasizes the complementary rather than interchangeable nature of both approaches (20). But although protocols for IP of endogenous membrane proteins are published (21–23) optimized protocols when dealing with low abundance proteins are lacking.

The receptor p75NTR is an excellent example of a transmembrane protein that poses a challenge to endogenous IP experiments with subsequent mass spectrometric detection. The molecular mass of p75NTR amounts to approx. 42 kDa based on the amino acid composition. The protein runs between 65 to 80 kDa in denaturing SDS-PAGE as it is subject to extensive post-translational modification with intra- and intermolecular disulfide-linkages, O- and N-glycosylation (24–28). In contrast to other membrane proteins, effective solubilization of p75NTR from cell membranes can be achieved by various buffers, including compositions based on detergents CHAPS (29, 30), NP-40 (31–33), n-octyl β-D-glucopyranoside (34, 35), Triton X-100 (36–39) and RIPA buffer (26, 40). Successful p75NTR IP from human urine samples confirms solubility even in detergent-free solutions (41, 42). Several p75NTR specific antibodies were generated and have been used extensively, e.g. 192-IgG (43), ME20.4 (44), and MLR1/2 (45). To our knowledge, however, there are only a few articles that report mass spectrometry after IP with antibodies detecting p75NTR epitopes. This approach was employed to confirm the successful capture of p75NTR itself (42), to investigate its phosphorylation sites (46) and to detect novel interacting proteins Galectin-3 and Trio (39, 47).

This comprehensive protocol collection details the workflow to successfully immunoprecipitate p75NTR at endogenous, low abundance expression levels. We validated the specificity of three monoclonal p75NTR antibodies (1 commercially available, 2 hybridoma culture derived) and IP conditions in human melanoma cell line A375, a model cell line for these conditions (9, 48). We then applied our experimental conditions to human leukemic plasmacytoid dendritic cell line PMDC05, a pDC-like cell line that had not been investigated for p75NTR expression before but – based on our data – shows very low endogenous p75NTR expression (49, 50). Since immune responses of dendritic cell subsets were shown to be modulated by low abundance levels of p75NTR (11, 12), we hypothesized that PMDC05 expressed p75NTR and could be employed as a pDC-like model cell line for p75NTR interaction experiments.

Our proposed workflow consisted of the following steps:

1. confirmation of p75NTR mRNA transcription by quantitative RT-PCR and protein expression by flow cytometry and immunofluorescence in both model and target cell lines
2. antibody validation in cell line A375 by IP, western blotting and mass spectrometry,
3. application of this optimized protocol to immunoprecipitate p75NTR and potentially interacting proteins in pDC-like cell line PMDC05.

## Materials and Equipment

### Cell Lines

- Mouse hybridoma cell line 200­3­G6­4 (20.4; HB-8737) producing p75NTR antibody ME20.4, American Type Culture Collection, Manassas, VA, USA
- Human melanoma cell line A375, a kind gift from Friedegund Meier and Dana Westphal, Clinic of Dermatology, University Clinic Carl Gustav Carus, Dresden, Germany
- Human leukemic plasmacytoid dendritic cell line PMDC05, provided by Miwako Narita (Laboratory of Hematology and Oncology, Graduate School of Health Sciences, Niigata University, Niigata, Japan), Kana Sakamoto and Kengo Takeuchi (both Cancer Institute of the Japanese Foundation for Cancer Research, Tokyo, Japan) (50)

### Cell Culture Reagents and Chemicals

Cell culture reagents and chemicals are detailed in **Supplementary Table S1**.

### Antibodies

Details about the use of antibodies in immunofluorescence and flow cytometry are listed in **Table 1**. P75NTR antibody MLR2 production has been described earlier (45). MLR2 antibody requests for academic and non-profit use may be addressed to Mary-Louise Rogers (mary-louise.rogers@flinders.edu.au). Unlabeled p75NTR antibody ME20.4 was produced in hybridoma cells. After cell expansion in IMDM supplemented with 10% FCS and 2 mM GlutaMAX, medium was switched to Hybridoma-SFM. Supernatant containing the antibody was stored at -20°C. Fluorescent dye-conjugated p75NTR antibodies ME20.4-1.H4 (PE), appropriate isotype control (IS5-21F5) and FcR human blocking reagent were purchased from Miltenyi Biotech. Clone D4B3 and isotype control DA1E, and anti-rabbit IgG-HRP were obtained from Cell Signaling Technology. Polyclonal rabbit IgG isotype control (NBP2-24891) was purchased from Novus Biologicals.

**Table 1:** Antibodies in immunofluorescence and flow cytometry.

Antibody	Clone	Host	Isotype	Function	Dye	Concentration [µg mL^-1^]	
						IF	FC
**p75NTR**	D4B3	rabbit	IgG	primary	unconjugated	0.1	0.1
	ME20.4	mouse	IgG1	primary	PE	0.8	–
	ME20.4	mouse	IgG1	primary	unconjugated	–	1.5
	MLR2	mouse	IgG2a	primary	unconjugated	2	2
**Isotype**	DA1E	rabbit	IgG	primary	unconjugated	–	0.1
	polyclonal	rabbit	IgG	primary	unconjugated	0.1	–
	eBM2a	mouse	IgG2a	primary	unconjugated	2	–
	DM1a	mouse	IgG1	primary	unconjugated	–	1.5
	IS5-21F5	mouse	IgG1	primary	PE	0.8	0.8
**2nd anti-mouse**	polyclonal	goat	IgG	secondary	AF555	4	–
	polyclonal	chicken	IgY	secondary	AF647	–	4
**2nd anti-rabbit**	polyclonal	goat	IgG	secondary	AF555	4	–
	polyclonal	chicken	IgY	secondary	AF647	–	4

AF555, Alexa Fluor 555; AF647, Alexa Fluor 647; FC, flow cytometry; IF, immunofluorescence; PE, phycoerythrin.

Goat anti-Rabbit IgG-Alexa Fluor 555 (A-21429), chicken anti-mouse IgG-Alexa-Fluor 647 (A-21463), goat anti-Mouse IgG-Alexa Fluor 555 (A-21424), Chicken anti-Rabbit IgG-Alexa Fluor 647 (A-21443) were purchased from Thermo Fisher Scientific. HRP-linked Donkey anti-Rabbit IgG (NA9340) was obtained from GE Healthcare.

### Recipes for Buffers and Media

- Elution Buffer: 0.1 M glycine in distilled water (pH 2.4 adjusted with HCl).
- Lysis Buffer: 130 mM NaCl, 50 mM HEPES (pH 7.4), 1% (v/v) Triton X-100 in distilled water. Supplemented with 1 tablet protease inhibitor cocktail (Roche cOmplete Ultra Mini EDTA-free) per 10 mL.
- 4% (w/v) PFA solution: dissolve 4% (w/v) PFA in HBSS, incubate at 80°C until solution becomes transparent, cool on ice. Store at 4°C and use for a month.
- HBSS: CaCl_2_ 0.14 g L^-1^, MgCl_2_ · 6 H_2_O 0.1 g L^-1^, MgSO_4_ · 7 H_2_O 0.1 g L^-1^, KCl 0.4 g L^-1^, KH_2_PO_4_ 0.06 g L^-1^, NaCl 8 g L^-1^, Na_2_HPO_4_ · 7 H_2_O 0.09 g L^-1^, D-glucose 1 g L^-1^ in ddH_2_O.
- PBS: KCl 0.2 g L^-1^, KH_2_PO_4_ 0.2 g L^-1^, NaCl 8 g L^-1^, Na_2_HPO_4_ 1.15 g L^-1^ in ddH_2_O.
- PBS++: 0.5% (w/v) BSA and 2 mM EDTA in PBS.
- PBS-DAPI: 0.1 µg mL^-1^ DAPI in PBS.
- Permeabilization Buffer: 0.1% (v/v) Triton X-100 and 1% (w/v) BSA in PBS.
- RPMI complete: RPMI 1640 supplemented with 10% (v/v) FCS, 2 mM GlutaMAX and 100 units mL^-1^ penicillin-streptomycin.
- SDS Sample Buffer (2×): 5% (w/v) SDS, 25% (v/v) glycerol, 20% (v/v) 2-mercaptoethanol, 0.25 mM Tris (pH 6.8) in ddH_2_O.
- TBS-T: 0.05% (v/v) Tween 20 in TBS (0.05 mM Tris, 0.145 M NaCl, pH 7.6, made from 10 × stock solution).
- TBS-T-M: 5% (w/v) nonfat dry milk powder in TBS-T.
- Washing Buffer: 130 mM NaCl, 50 mM HEPES (pH 7.4, adjusted with HCl).
- 10× Tris buffered saline (500 mM Tris, 1.45 M NaCl, pH 7.6).

## Methods

### Biosafety Notes:

- Both cell lines A375 and PMDC05 must be handled at biosafety level 1: wear personal protective equipment (gloves, lab coat, protective eyewear if splashes are possible) at all times.
- Adhere to additional biosafety regulations of your institution, especially when transfected or infectious cell lines are used.

### Protocols for step 1—Confirmation of p75NTR Expression by Quantitative RT-PCR, Flow Cytometry and Immunofluorescence in Cell Lines A375 and PMDC05

#### Culture of Human Melanoma Cell Line A375

1. Cultivate at a density of 2-5 × 10^4^ cells cm^-2^ in pre-warmed RPMI complete (0.2 mL cm^-2^) in an incubator (37°C, 95% humidity, 5% CO_2_).
2. Passaging (usually after 2-3 days)

a. remove cell culture supernatant, gently add PBS (0.1 mL cm^-2^), swivel cell culture vessel and discard supernatant.
b. add pre-warmed 21 µM trypsin solution (0.03 mL cm^-2^) and incubate at 37°C for 5 min, check cell detachment with microscope.
c. add 3 volumes of RPMI complete.
d. transfer cell suspension to a centrifuge tube and centrifuge at 300 × g for 8 min at RT.
e. resuspend cells in pre-warmed RPMI complete and count cells (e.g. in a Neubauer chamber).
f. adjust cells to desired concentration.

#### Culture of Human Leukemic Plasmacytoid Dendritic Cell Line PMDC05

1. Cultivate at a density of 0.25-0.5 × 10^6^ cells cm^-2^ in pre-warmed RPMI complete (0.2 mL cm^-2^) in an incubator (37°C, 95% humidity, 5% CO_2_).
2. Passaging (usually twice a week)

a. transfer cell suspension to a centrifuge tube, rinse surface of the culture vessels with PBS (0.05 mL cm^-2^) to loosen slightly attached cells and centrifuge at 300 × g for 8 min at RT.
b. resuspend cells in pre-warmed RPMI complete and count cells (e.g. in a Neubauer chamber).
c. Adjust cells to desired concentration.

##### Critical Parameters

- Viable cell concentration can be determined by trypan blue exclusion.
- PMDC05 are sensitive, slowly growing cells and tend to form clusters. At the beginning, they may struggle to regenerate from the thawing process. Once stable growth has been observed subcultivation intervals may be increased to 7 days.

#### Sample Preparation for RNA Isolation and Whole Cell Protein Lysates

1. Transfer cells to centrifuge tubes and perform the following steps at 4°C.
2. Wash cells twice by centrifugation at 300 × g for 8 min and resuspension in ice-cold 1x PBS.
3. For RNA preparation:

a. Transfer 5 × 10^6^ cells to a 1.5 mL reaction tube.
b. Centrifuge at 500 × g for 8 min.
c. Carefully remove supernatant as completely as possible.
d. Store dry pellets at -80°C.
4. For whole cell protein lysates:
a. Transfer 2-10 × 10^7^ cells to a 1.5 mL reaction tube.
b. Lyse cells in lysis buffer (10^8^ cells mL^-1^) e.g. by trituration.
c. Incubate for 1 hour under constant agitation.
d. Clear lysates by centrifugation at ≥10,000 × g for 45 min.
e. Store lysates at -20°C.

#### RNA Isolation, cDNA Synthesis, and Quantitative Real-Time PCR

Total RNA was purified from human cell lines A375 and PMDC05 using RNeasy Mini Kit (QIAGEN) according to the manufacturer’s instructions. Content and purity of RNA preparations was assessed on a microvolume spectrometer (NanoDrop One, Thermo Fisher Scientific) by calculating 260 nm/280 nm absorbance ratios. Complementary DNA (cDNA) was synthesized using SuperScript IV First-Strand Synthesis System (Thermo Fisher Scientific) with oligo-dT primers according to the manufacturer’s instructions. Quantitative PCR was performed on a QuantStudio 5 Real-Time-PCR-System (Thermo Fisher Scientific) using the TaqMan system (Thermo Fisher Scientific) according to the manufacturer’s instructions. Assay probes were purchased from Thermo Fisher Scientific designed to cover exons 1-5 of human p75NTR (NCBI Reference Sequence: NM_002507.3; for details see **Supplementary Table S2**).

#### Fluorescent Dye Labeled Antibody Staining for Flow Cytometry

1. Transfer cell suspension prepared during cell culture handling to a centrifuge tube.
2. Perform the following steps at 4°C.
3. Centrifuge at 300 × g for 8 min and resuspend in ice-cold PBS++.
4. Repeat the centrifugation step and resuspend in PBS++ containing human FcR blocking reagent (1:10) at a concentration of 2 × 10^7^ cells mL^-1^.
5. Transfer 50 µL of cell suspension to a 96-well plate cavity.
6. Incubate for 10 min.
7. Add 200 µL PBS++, centrifuge for 8 min at 300 × g and discard the supernatant.
8. Resuspend in 55 µL PBS++ containing the appropriate antibody concentration (see **Table 1**).
9. Incubate for 30 min in the dark.
10. Add 200 µL PBS++, centrifuge for 8 min at 300 × g and discard the supernatant.
11. If using fluorescent dye labeled primary antibodies, proceed to step 13.
12. If using primary-secondary staining:

a. Wash cells again with 200 µL PBS++.
b. Resuspend in 55 µL PBS++ containing the appropriate secondary antibody (see **Table 1**).
c. Incubate for 30 min in the dark.
d. Add 200 µL PBS++, centrifuge for 8 min at 300 × g and discard the supernatant.
13. Resuspend in 200 µL PBS++.
14. Transfer to a FACS tube and add an equal amount of PBS-DAPI resulting in a working DAPI concentration of 0.05 µg µL^-1^.
15. Flow cytometry was performed on an LSR II flow cytometer (BD Biosciences). Data were analyzed using FlowJo (Version 10.6.1, BD Biosciences).

##### Critical Parameters

- An important step when handling immune cells for flow cytometry consists in blocking receptors that may bind Fc parts of immunoglobulins. FcR blocking reagents should always be included to prevent unspecific antibody binding.
- Remember to include unstained and isotype controls.
- When staining with multiple dyes, compensation for interfering fluorescence signals is recommended.
- Typical DAPI working concentrations range from 0.05-2 µg µL^-1^. Although general recommendations on the use of DAPI in flow cytometry exist, working concentrations should be validated in the specific experimental condition.
- Based on their fluorescence intensity, different cell subsets can be sorted [for suitable protocols see e.g. (51, 52)]. This approach may be used to enrich populations with certain expression levels of the protein of interest. The success rates of subsequent IP experiments may increase because the depletion of non-expressing cells reduces the background noise.

#### Immunofluorescence

1. Coat sterile precision coverslips (Carl Roth) with poly-L-lysine (Merck)

a. Add 0.01% (v/v) poly-L-lysine solution to coverslips (40 µL cm^-2^).
b. Incubate for 5 min at room temperature on a rocking platform.
c. Remove poly-L-lysine solution and rinse with double distilled water.
d. Allow coated coverslips to dry for at least 2 hours under a sterile hood before further use.
2. Grow cells overnight on poly-L-lysine coated coverslips with at a density of 5 × 10^4^ A375 cells cm^-2^ or 1 × 10^5^ PMDC05 cells cm^-2^.
3. Wash A375 coverslips three times by gentle addition of HBSS (containing calcium, magnesium)
a. PMDC05 coverslips were centrifuged in 24-well plates at 300 × g for 5 min at each washing step.
4. Fix cells in 4% (w/v) PFA solution for 15 min at 37°C.
5. Remove 4% (w/v) PFA solution and wash cells three times for 5 min with HBSS on a shaker.
6.Counterstain the membrane with WGA (wheat germ agglutinin; 5 µg mL^-1^) in HBSS for 10 min at 37°C.
7.Wash A375 cells three times in HBSS for 5 min on a rocking platform at medium speed.
a. Wash PMDC05 cells three times by centrifugation in 24-well plates at 300 × g for 5 min.
8.*Optional permeabilization step:*
a. Incubate cells for 15 min in Permeabilization Buffer.
b. Remove Permeabilization Buffer carefully.
9. Block A375 cells for 60 min in 1% (w/v) BSA in PBS and PMDC05 cells for 60 min in 1% (w/v) BSA in PBS containing human FCR Block (1:80), and 10% goat/mouse serum (depending on the dye-labeled primary and secondary antibody).
10. Stain cells with the primary antibodies in 1% (w/v) BSA in PBS.
11. Incubate for 2 hours (A375) at RT or overnight (PMDC05) in a wet chamber in the dark at 4°C.
12. Wash A375 cells three times in HBSS for 5 min on a rocking platform at medium speed.
a. Wash PMDC05 cells three times by centrifugation in 24-well plates at 300 × g for 5 min.
13. Stain cells with the secondary antibody in 1% (w/v) BSA in PBS.
14. Incubate for 60 min at RT in a wet chamber in the dark.
15. Wash A375 cells three times in HBSS for 5 min on a rocking platform at medium speed.
a. Wash PMDC05 cells three times by centrifugation in 24-well plates at 300 × g for 5 min.
16. Counterstain DNA with 1 µg Hoechst 33342 mL^-1^ in HBSS for 15 min at 37°C.
17. Wash A375 cells twice in HBSS for 5 min and once in distilled water on a rocking platform at medium speed.
a. Wash PMDC05 cells twice in HBSS and once in distilled water by centrifugation in 24-well plates at 300 × g for 5 min.
18. Remove coverslips carefully and mount them with VECTASHIELD Antifade Mounting Medium on glass slides.

Microscopy was performed according to Weidemuth et al. (53). Briefly, stained cells were imaged with a Leica SP5 inverse microscope (Leica). Confocal images were collected at 405, (488), 543 and 594 nm with a 63 × NA1.4 objective lens. Image acquisition, shutter, Z-axis position, laser lines, and confocal system were all controlled by Leica LAS AF software. Equivalent exposure conditions were used between samples. Depending on cell density, a digital zoom was applied to present numerous cells per image. Images were analyzed using Fiji software (54). Image processing includes a median filter with a radius of 1 pixel to subtract background, and equal brightness and contrast adjustments between the samples.

##### Critical Parameters

- PMDC05 cells basically grow in suspension and therefore adhere to coated coverslips weakly. To avoid losing cells during the staining procedure shortened washing and centrifugation is mandatory. That might affect cell integrity and thus antibody localization. The risk of losing adherent A375 cells after fixation is minimal.
- Permeabilization and fixation chemicals might influence epitope accessibility and therefore antibody binding. Different protocols for permeabilization and fixation [e.g. (55)] may be tried to achieve optimal results.

### Protocols for Steps 2 and 3—Immunoprecipitation and Analysis by Mass Spectrometry and Western Blotting

#### Immunoprecipitation

1. Perform all steps on ice/at 4°C (unless indicated otherwise).
2. Resuspend 1.5 mg magnetic protein G Dynabeads in PBS in 1.5 mL reaction tube.
3. Wash beads in following procedure

a. Put on a static magnet (e.g. DYNAL MPC-S, Thermo Fisher Scientific), collect beads by incubation for 5 min.
b. Carefully aspirate supernatant and discard without disturbing collected beads.
c. Remove bead containing reaction tube from static magnet.
d. Resuspend beads by gently pipetting up and down in 500 µL PBS and repeat washing steps from a to c twice.
4. For antibodies MLR2 and D4B3:
a. Prepare 200 µL PBS containing 2 µg D4B3 and 10 µg MLR2, respectively.
b. resuspend beads by gently pipetting up and down in antibody coupling solution.
5. For antibody ME20.4:
a. resuspend beads gently in 10 mL serum free hybridoma culture supernatant.
6. Prepare coupling solutions containing the same concentration of isotype control antibody.
a. resuspend beads by gently pipetting up and down in antibody coupling solution.
7. Incubate magnetic beads for 2 h at room temperature under constant agitation to prevent the beads from settling down.
8. Put on a static magnet, collect beads by incubation for 5 min.
9. Carefully aspirate supernatant and discard.
10. Remove bead containing reaction tube from static magnet.
11. Add 200 µL protein lysates (corresponding to 2 × 10^7^ cells) and adjust volume to 500 µL by adding lysis buffer, if necessary.
12. Incubate overnight under constant agitation.
13. Put on a static magnet, collect beads by incubation for 5 min.
14. Carefully aspirate supernatant and discard.
15. Wash with lysis buffer
a. Remove bead containing reaction tube from static magnet.
b. Resuspend beads by gently pipetting up and down in 500 µL lysis buffer.
c. Put on a static magnet, collect beads by incubation for 5 min.
d. Carefully aspirate supernatant and discard.
16. Wash with washing buffer
a. Remove bead containing reaction tube from static magnet.
b. Resuspend beads by gently pipetting up and down in 500 µL washing buffer.
c. Put on a static magnet, collect beads by incubation for 5 min.
d. Carefully aspirate supernatant and discard.
e. repeat steps a to d once.
17. Remove bead containing reaction tube from static magnet.
18. Resuspend beads in 100 µL elution buffer.
19. Incubate at room temperature under constant agitation for 10 min.
20. Put on a static magnet, collect beads by incubation for 5 min.
21. Carefully aspirate and save eluate (fraction E1).
22. For a second elution (fraction E2), repeat steps 17-21.
23. Resuspend beads in 100 µL SDS sample buffer.
24. Heat to 95°C and incubate for 5 min under constant agitation.
25. Put on a static magnet, collect beads by incubation for 5 min and save eluate (fraction col).

##### Critical Parameters

- Careful resuspension of magnetic beads ensures the bead integrity. But especially after coupling antibodies, harsh treatment, e.g. vortexing, must be avoided to preserve stable binding and capture.
- Addition of detergent to all washing solutions and coupling buffers prevents clumping of beads and may reduce unspecific binding if downstream applications permit this.
- For mass spectrometry, any detergent must be washed out before analysis. This is ensured by washing and elution in detergent-free buffer.
- If captured protein amounts are low, consider washing in detergent-free buffers. The use of crosslinking agents, e.g. dithiobis succinimidyl propionate, is compatible with many downstream applications.
- Heating protein samples is a critical step when eluting in SDS sample buffer. Hydrophobic membrane proteins may precipitate when heated above 70°C. Avoid long incubation times at high temperatures as peptide bonds may hydrolyze.

#### SDS Polyacrylamide Gel Electrophoresis, Western Blotting and Membrane Staining

1. Add 10 µL 2× SDS sample buffer to 10 µL sample solution.
2. Incubate at 95°C for 10 min under constant agitation.
3. Load samples and protein standard on gels and run electrophoresis for 35 min keeping voltage constantly at 200 V.
4. Transfer gel proteins to Immobilon-P transfer membrane in a wet chamber for 90 min keeping voltage constantly at 30 V.
5. Perform all following steps under constant agitation (e.g. on a rocking platform).
6. Wash membrane three times for 5 min in 20 mL TBS-T.
7. block membrane in 20 mL TBS-T-M for 15 min at room temperature.
8. Incubate with D4B3 antibody buffer (1:1000 in 4 mL TBS-T-M) overnight at 4°C.
9. Wash membrane three times for 5 min in 20 mL TBS-T.
10. Incubate in secondary antibody solution (anti rabbit-HRP, 1:10,000 in 10 mL TBS-T-M) for 1 hour at room temperature.
11. Wash membrane three times for 5 min in 20 mL TBS-T.
12. Add Lumi-Light PLUS western blotting substrate (10-15 µL cm^-2^) to PVDF membrane.
13. Detect signal on the Azure c600 imaging device (Azure Biosystems).

##### Critical Parameters

- Addition of dyes to SDS sample buffers may ease loading protein samples onto gels.
- Some proteins may be heat-sensitive (see above). Lower temperatures if necessary.
- Larger proteins transfer from gel to membrane more slowly than smaller ones. Protein transfer may be assessed by gel staining with Coomassie or silver-based dyes. Transfer times may be extended if protein transfer is insufficient. Membranes with small pore sizes (0.22 µm) may be sensible when smaller proteins are lost during transfer.
- If capture and detection antibody are derived from the same species HRP-linked protein A/G may be used instead of a secondary antibody. This approach minimizes unspecific detection of denatured capture antibodies (**Figure 3**).
- A positive control is recommended when sampling unknown antibodies in western blot. These positive controls can be manufactured from cell lines/tissues with a well detectable expression of the protein in question. In our case, an example is cell line A875. If such a cell line or tissue is not available an alternative strategy is the generation of a cell line with a transgenic expression (see **Supplementary Methods**).

#### Mass Spectrometry

1. For information on the instrumentation and software applied see **Table 2**.
2. Resuspend beads in 100-200 µL suitable buffer for proteolytic digestion (e.g. 20-50 mM Tris-HCl or HEPES pH 7.5).
3. Add 2 µL sequencing grade Trypsin (100 ng µL^-1^) and incubate overnight (12-18h) at 37°C in a shaker to avoid settling of the beads.
4. Add additional 2 µL sequencing grade Trypsin (100 ng µL^-1^) and incubate again overnight at 37°C in a shaker.
5. Add 2 µL sequencing grade Lys-C (e.g. 50 ng µL^-1^ for rLys-C from Promega) and incubate again overnight at 37°C in a shaker.
6. Spin-down beads and collect supernatant.
7. Acidify supernatant with 2% TFA (pH<2) and desalt on C18 ultramicro column (e.g. Nest Group or Harvard Scientific Ultramicro Spin-Columns C18) according to the manufacturer’s instructions (61).
8. Dry the eluate of the desalting column in a speed vac and store the dry peptide mixture at -20°C until LC-MS/MS analysis.
9. Dissolve the peptide mixture in an appropriate volume formic acid (final concentration 3.5-4.0%) and transfer the solution into an HPLC vial for LC-MSMS analysis.
10. Proteomic LC-MS/MS is commonly performed with a nanoflow UPLC system hyphenated directly to a mass spectrometer. Low flow rates of 200-300 nL min^-1^ and linear gradients of water, 0.1% formic acid (A) and acetonitrile, 0.1% formic acid achieve the high sensitivity recommended for this kind of analysis. Commonly the mass spectrometer is operated in data dependent acquisition mode selecting the most intense peptide ions for fragmentation automatically.
11. The peptide fragmentation data is extracted from the raw MS data files and submitted to a data interpretation program that identifies the peptides and the proteins present in the sample.
12. Quantification of proteins can be performed to gain insight into quantitative changes between samples rather than judgment by presence or absence of proteins to differentiate true interactors from experimental background.
13. To support data interpretation the enrichment of protein identifications with additional information, e.g. GO molecular function or localization, may prove helpful and suitable commercial and academic programs are available.

**Table 2:** Mass spectrometry: instrumentation and software.

	Parameters / Comments	Vendor
**Dionex3000 RLSC UPLC system**	flow rate 200nl/min	ThermoScientific
****	linear gradient 0%-60% B in 90 min	
	(A: water, 0.1% formic acid, B: 60% acetonitrile, 40% water, 0.1% formic acid)	
	Columns: PepMap Acclaim C18	
**Q-Exactive HF mass spectrometer**	DDA Acquisition mode, Top10	ThermoScientific
****	Resolution: MS1 60000, MS2 15000	
**MASCOT (peptide and protein identification)**	MS1: 10 ppm	Matrixscience
****	MS2: 30 mmu	matrixscience.com
****	Variable modifications: protein N-acetyl, methionine oxidation	(56, 57)
**MS Convert (file conversion to mgf)**	part of the ProteoWizard toolbox	ProteoWizard
****	Version 3.0.19096-68e50d059	proteowizard.com (58)
		
**Scaffold (Validation of protein/peptide identification, visualization, GO annotation)**	Version 4.11.1	Proteome Software Inc.
****		proteomesoftware.com (59, 60)
**Progenesis QI for Proteomics (protein quantification)**	Version 4.2	Nonlinear Dynamics
****		nonlinear.com

##### Critical Parameters

- The protease quality is essential for efficiency and specificity of the digestion. Sequencing grade proteases are recommended.
- Trypsin is generally available as stabilized (chemically modified) enzyme that reduces auto-digestion of the enzyme.
- Lys-C is available as native and recombinant enzyme, and the specific activity (U mg^-1^) may vary depending on the manufacturer.
- Speed of the shaker should be just intense enough to keep the beads in suspension.
- Proteomic LC-MS/MS is commonly performed with nanoflow UPLC system operated at flow rates of 200-300 nL min^-1^ with linear gradients of water, 0.1% formic acid (A) and acetonitrile, 0.1% formic acid. Different companies offer this kind of LC systems and specific parameters for equilibration time, gradient steepness and handling may apply.
- Different mass spectrometers are suitable for mass spectrometric analysis of complex peptide mixtures and differ in resolution, acquisition speed and peptide fragmentation methodology. In general, a typical approach for the bottom-up proteomic LC-MS/MS analysis is the fragmentation of the most intense multiply charged (z = +2, +3, …, +5) peptide ions (TopN) in data-dependent acquisition mode.
- Peptide and protein identification from mass spectrometric raw data requires extraction of fragment spectra, matching of experimental spectra with theoretical spectra or spectral libraries and a variety of academic and commercial software are available (56, 59, 60, 62).

## Results

### Step 1—p75NTR Expression by TaqMan and p75NTR Detection by Antibodies D4B3, ME20.4 and MLR2

A prerequisite for the analysis of protein-protein interactions is the detection of p75NTR expression on RNA and protein level. The p75NTR gene has six exons, as annotated in the reference transcript NM_002507.3 (63). We used four different TaqMan probes covering the exon boundaries 1-2, 2-3, 3-4, and 4-5, and one covering *gapdh* for quantification of RNA expression. All analyzed exons are expressed in cell lines A375 and PMDC05 with an average expression compared to *gapdh* of 0.26 and 0.29%, respectively, indicating a low expression in both cell lines.

**Figure 1:**
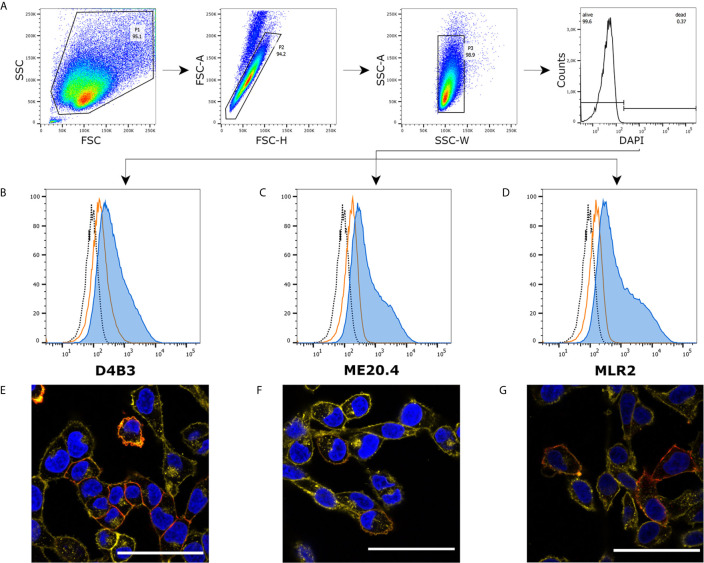
Immunofluorescence and flow cytometry of A375 cells. **(A)** Recommended gating strategy with a double singlet gate and a DAPI labeled living/dead gate. **(B–D)** D4B3, ME20.4, MLR2 staining analyzed by flow cytometry (**▪** unstained cells, **▪** isotype control stained cells, **▪** p75NTR stained cells). **(E–G)** Immunofluorescence microscopic images of D4B3, ME20.4, MLR2 stains (**▪** nuclear staining with DAPI, **▪** p75NTR staining, **▪** membrane staining with WGA-AF594). Scale bar = 50 µm.

We investigated expression on protein level in melanoma cell line A375 using three different p75NTR specific antibodies, D4B3 (rabbit IgG), ME20.4 (mouse IgG1) and MLR2 (mouse IgG2a). Cells were prepared and labeled for flow cytometry according to protocol. In a strict gating strategy with two singlet gates and a DAPI-based dead cell exclusion gate (**Figure 1A**), each antibody exhibited a specific staining pattern compared to unstained and isotype control labeled cells (**Figures 1B–D**). The shoulder in p75NTR graphs hints at a distinct A375 population with increased p75NTR antibody binding. Immunofluorescence microscopy revealed that only a subset of A375 cells stained positive for p75NTR (**Figures 1E–G**). The ME20.4 staining (**Figure 1F**) appeared slightly fainter in comparison with D4B3 and MLR2 stainings (**Figures 1E, G** **)**. Isotype controls and single channel images as well as images of permeabilized cells are provided in the supplementary information (**Figures S1**, **S2**).

**Figure 2:**
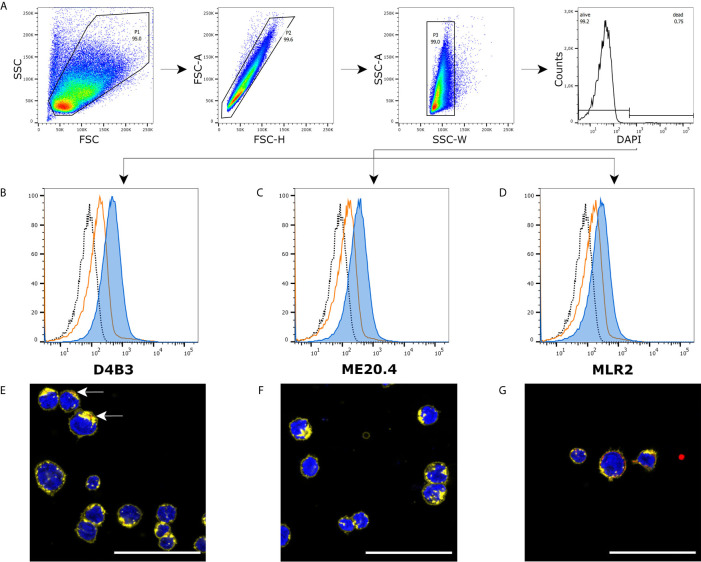
Immunofluorescence and flow cytometry of PMDC05 cells. **(A)** Recommended gating strategy with a double singlet gate and a DAPI labeled living/dead gate. **(B–D)** D4B3, ME20.4, MLR2 staining analyzed by flow cytometry (**▪** unstained cells, **▪** isotype control stained cells, **▪** p75NTR stained cells). **(E–G)** Immunofluorescence microscopic images of D4B3, ME20.4, MLR2 stains (**▪** nuclear staining with DAPI, **▪** p75NTR staining, **▪** membrane staining with WGA-AF594). **(E)** The arrows point towards a faint p75NTR signal. Scale bar = 50 µm.

The same approach was chosen to analyze p75NTR expression in pDC-like cell line PMDC05. Applying the same gating strategy (**Figure 2A**), all three antibodies detected a specific signal in this cell line (**Figures 2B–D**). In comparison to A375 cells, the prominent shoulder in the graphs is missing and median fluorescent intensity is lower but different from controls. Immunofluorescence microscopy is rather challenging due to the non-adherent nature of PMDC05. An additional centrifugation step after each washing was included in our protocol. D4B3 exhibited a faint signal (**Figure 2E**). ME20.4 staining resulted in no detectable signal (**Figure 2F**). Only, MLR2 was able to provide a stronger signal (**Figure 2G**). The corresponding isotype control stainings, single channel images, and images of permeabilized cells are shown in **Figures S3** and **S4**.

Summarizing our findings, both cell lines expressed low levels of p75NTR mRNA. On protein level, PMDC05 cells appeared to express even less p75NTR than A375 cells. Compared to cell lines with no and high p75NTR expression (see **Supplementary Information** and **Figure S5**), the observed slight increases in fluorescence intensity do correspond to low p75NTR expression. This conclusion was drawn after careful interpretation of expression data from all cell lines (for details see **Supplementary Results**).

### Step 2—Validation of Antibodies and IP Conditions in Model Cell Line A375

**Figure 3:**
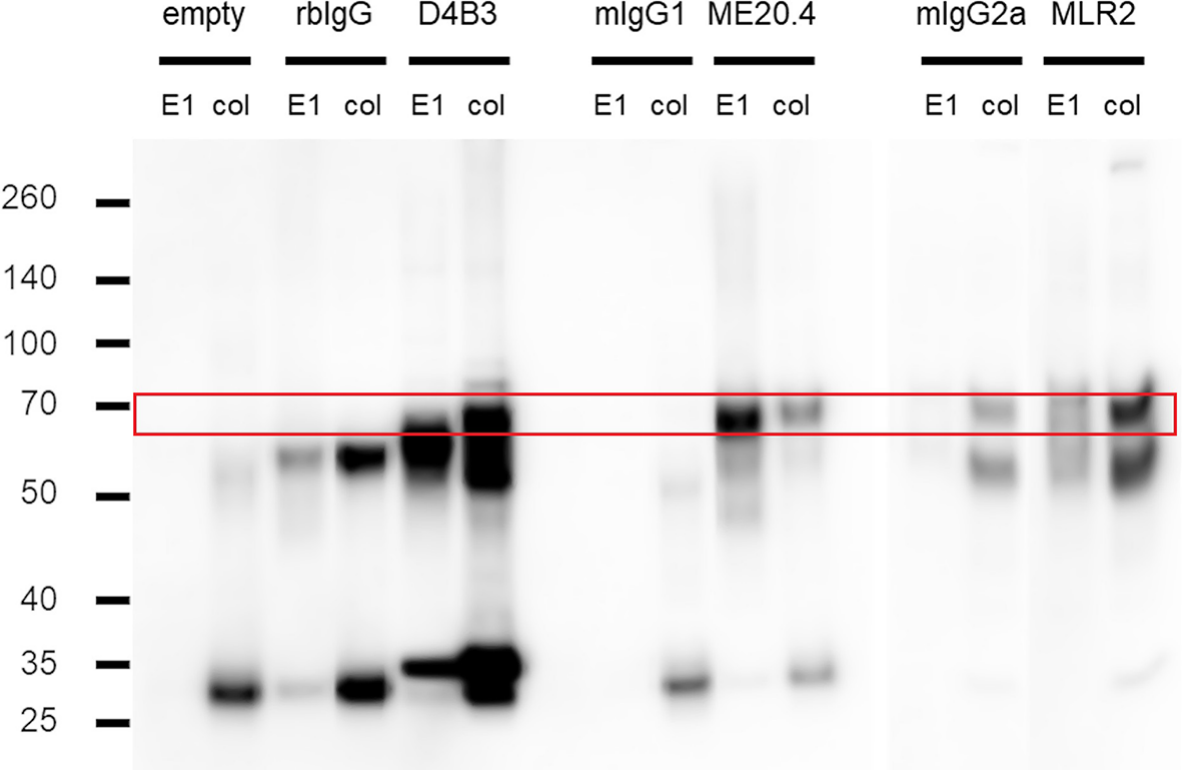
Immunoprecipitation experiments in A375. Western blots of acid (E1) and sample buffer (col) elution fractions from IP trials with D4B3, ME20.4 and MLR2, respective isotype matched (rbIgG, mIgG1, mIgG2a) and empty controls. P75NTR band runs just below 70 kDa (red box) as can be seen faintly in D4B3-E1, ME20.4-col and MLR2-E1. More intense bands are observed in D4B3-col, ME20.4-E1 and MLR2-col. Heavy chains run at approx. 55 kDa (especially prominent in rbIgG lanes), light chains just above 25 kDa. Figure is composed of different western blot membranes. *Detection:* primary antibody D4B3, secondary antibody anti-rabbit IgG-HRP (GE).

For this purpose, lysates from 2 × 10^7^ A375 cells were prepared and used for immunoprecipitation experiments with antibodies D4B3, ME20.4 and MLR2. Immunoprecipitation was performed according to protocol. Great effort was put into gentle but complete resuspension of the beads. Acid elution and SDS sample buffer elution fractions were loaded on a gel for SDS PAGE and subsequently transferred to a Immobilon-P transfer membrane by western blotting. Membrane staining was carried out with p75NTR antibody D4B3 due to its superior performance (**Figure 3**). Acid elution fractions (E1) from all IPs exhibited a signal just below 70 kDa (marked by the red box) that was not detected in the empty (no antibody) and isotype control experiments (rbIgG, mIgG1, mIgG2a). A similar pattern was detected in SDS sample buffer elutions (col) from D4B3 and ME20.4 IPs. The MLR2 sample buffer elution fraction exhibited a strong band pattern with a characteristic band just below 70 kDa. A corresponding band, although with significantly less intensity, was detected in mIgG2a isotype control experiments. Non-specific antibody-mediated interactions to other proteins or the bead matrix may cause the faint band pattern.

To verify the specific binding by a second method (and to avoid circular reasoning), first elution fractions (in 0.1 M glycine, pH 2.4) from the same immunoprecipitations were analyzed by mass spectrometry. Given the amino acid sequence of p75NTR, its extensive post-translational modifications and our protein digestion techniques, the number of reliably detectable peptides is limited. Theoretical prediction resulted in four detectable peptides. Expert opinion excluded one based on an extremely low detection probability due to its length. All three p75NTR specific peptide sequences were detected in acid elution fractions from immunoprecipitations with antibodies D4B3, ME20.4 and MLR2 (**Table 3**) but not in acid elution fractions from isotype matched controls.

**Table 3:** Peptides detected by mass spectrometry.

Cell line	Antibody	Protein	Sequences	Mascot Score	Probability
**A375**	D4B3	p75NTR	(K)QGANSRPVNQTPPPEGEK(L) (K)GDGGLYSSLPPAK(R) (K)LLNGSAGDTWR(H)	31.1 58.9 57.1	100% 100% 100%
**A375**	ME20.4	p75NTR	(R)VCEAGSGLVFSCQDK(Q) (R)CAYGYYQDETTGR(C)	56.9 31.1	100% 100%
**A375**	MLR2	p75NTR	(K)QGANSRPVNQTPPPEGEK(L) (K)LLNGSAGDTWR(H) (K)GDGGLYSSLPPAK(R)	20.0 79.0 63.1	99% 100% 100%
**PMDC05**	ME20.4	p75NTR	(K)LHSDSGISVDSQSLHDQQPHTQTASGQALK(G) (K)GDGGLYSSLPPAK(R) (K)LLNGSAGDTWR(H)	12.4 69.8 41.9	95% 100% 100%
**PMDC05**	ME20.4	14-3-3ϵ	(R)YLAEFATGNDR(K) (K)EAAENSLVAYK(A)	11.7 30.3	97% 100%

In summary, we have shown that all three antibodies detect p75NTR specifically and reliably in low abundance model cell line A375. Due to the very specific band pattern of ME20.4, we chose the combination of ME20.4 for capture and D4B3 for detection antibody in step 3.

### Step 3—IP of p75NTR and Potentially Interacting Proteins in pDC-Like Cell Line PMDC05

**Figure 4:**
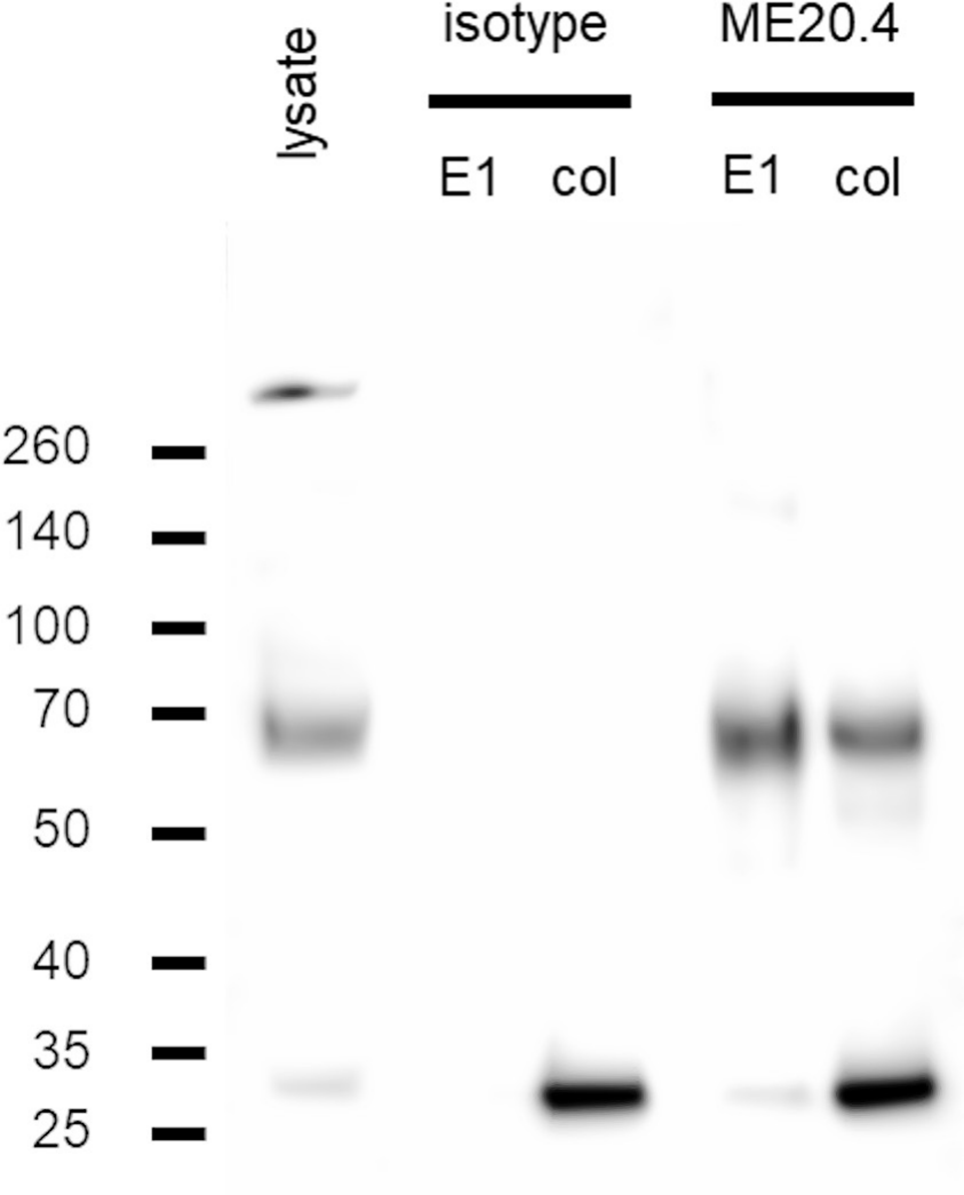
Immunoprecipitation experiments in PMDC05. Western blot of lysate, acid (E1) and sample buffer (col) elution fractions from IP trials with ME20.4 and an isotype matched (mIgG1) control. P75NTR band runs just below 70 kDa as can be seen in lysate. A specific p75NTR signal is detected in both ME20.4-E1 and ME20.4-col fraction. The band above 260 kDa in the lysate fraction could represent an incompletely split p75NTR oligomer.

The conditions validated in step 2 were applied to experiments with PMDC05. Again, lysates from 2 × 10^7^ cells were prepared. IPs were performed using the ME20.4-D4B3 antibody combination and the resulting western blots are shown in **Figure 4**. In a dilution experiment, specific p75NTR signals were still observed from a PMDC05 lysate input corresponding to 10^6^ cells (**Figure S6**). The acid elution fraction was analyzed by mass spectrometry which confirmed the specific detection of p75NTR (**Table 3**). Furthermore, 14-3-3ϵ was revealed as a potential interaction partner (**Table 3**). The interaction of p75NTR and 14-3-3ϵ has been described earlier (64).

In summary, we applied our IP conditions successfully to a cell line with a very low p75NTR expression and showed the potential of our setup to identify potentially interacting proteins.

## Discussion

When analyzing protein-protein interactions under endogenous, low abundance expression conditions the highest challenge is to filter out a specific signal from unspecific background noise. The present protocol collection provides the workflow to successfully immunoprecipitate transmembrane receptor p75NTR at endogenous, low abundance expression levels. Detailed step-by-step instructions guide through the complex process that leads to reliable and replicable results. After confirmation of p75NTR expression, antibodies D4B3, ME20.4 and MLR2 were validated for low abundance IP settings using human melanoma cell line A375 as a model cell line with known p75NTR expression levels. Under these conditions, rabbit monoclonal antibody D4B3 for western blot detection and murine monoclonal antibody ME20.4 for IP turned out the best combination. This experimental setup was successfully applied to human leukemic plasmacytoid dendritic cell line PMDC05 which has not been characterized regarding p75NTR expression before. Based on the direct comparison with cell line A375, which exhibits only a low p75NTR expression (9), we have shown that pDC-like cell line PMDC05 expresses p75NTR at an extremely low level. This extremely low expression appears to lie just above the detection limit as evidenced by the low but specific signals in flow cytometry and the fact that only one antibody, MLR2, exhibited a strong signal in immunofluorescence microscopy. Under these challenging conditions, we successfully immunoprecipitated p75NTR as confirmed by two independent methods: western blot detection with a different antibody and mass spectrometry. Furthermore, this approach specifically identified the p75NTR interactor 14-3-3ϵ as a probably interacting protein, in line with earlier findings (64). This proves the capability of our approach to provide new insights into p75NTR interactions, even at extremely low expression levels.

IP is a powerful straightforward method to investigate protein-protein interactions. As part of our strategy, we used the direct mass spectrometry analysis of acid elution fractions. To our knowledge, this has not been published in the context of p75NTR before. Other groups employing a combined IP/mass spectrometry approach analyzed proteins extracted from gel bands (39, 42, 46, 47). An alternative strategy is direct enzymatic digestion on the beads (65, 66). A necessary step in our approach is the elimination of detergent during washes and elution as these substances interfere with the downstream mass spectrometry protocol. This is in contrast to other protocols (67, 68). If the use of detergents is unavoidable these must be removed, e.g. by 1- or 2-dimensional SDS-PAGE (68). Alternative strategies depend on the detergent in question. In case of SDS, precipitation with sodium chloride has also been shown to effectively remove the detergent (69).

Apart from these technical and method-associated aspects, there may be challenging characteristics of investigated protein itself. The neurotrophin receptor p75NTR is an excellent example of such a protein. P75NTR is subject to extensive post-translational modification with intra- and intermolecular disulfide-linkages as well as O- and N-glycosylation (25–28). These modifications may limit the number of reliably detectable peptide sequences in mass spectrometry. Glycosylated protein domains promote hydrophilic interactions with carbohydrate-based bead matrices (70). These inherent characteristics render IP at native, low expression levels challenging. In contrast to IPs of recombinant proteins *via* their respective tag, IPs of endogenous proteins rely on antibodies binding to epitopes located within the protein itself. This very epitope, however, may serve as a binding site for ligands, interaction or oligomerization partners. In our case, all three antibodies bind to epitopes of the extracellular domain (44, 45). It has also been shown that ME20.4 and MLR2 may inhibit NGF binding at least partially. ME20.4, however, may immunoprecipitate p75NTR in complex with NGF after ligand binding, and is suitable to investigate ligand-mediated changes in the receptor interactome (44). Another determinant of successful IP experiments is the stability of protein complexes. Optimal experimental conditions may vary with antibodies and target proteins and must be chosen carefully to preserve intact complexes. Otherwise, interaction partners may not be detected.

The combination of immunoprecipitation and mass spectrometry can be employed in both confirmatory and exploratory way (71). If expression levels are presumed to directly influence protein interactions—as is the case for p75NTR—an investigation of these interactions under endogenous conditions is a logical approach. Even in challenging systems like plant extracts, the analysis in low abundance conditions identifies new interactors (72), highlighting the importance of our approach.

Our workflow provides the basis for reproducible and reliable research even in extremely low expression conditions. The optimized IP protocol lays the cornerstone for research in primary cells with low endogenous p75NTR expression like pDC, thereby improving our ability to listen to the whispers of physiological neuroimmune crosstalk and the subtle regulation of immune responses.

## Data Availability Statement

The original contributions presented in the study are included in the article/**Supplementary Material**. Further inquiries can be directed to the corresponding author.

## Author Contributions

BD performed most of the experiments. RW performed immunofluorescence staining and contributed to the manuscript. A-CF performed RNA isolation and quantitative RT-PCR. MG performed mass spectrometry experiments and contributed to the manuscript. M-LR prepared antibodies and contributed to the manuscript. SB and ST supervised experiments and contributed to the manuscript. All authors contributed to the article and approved the submitted version.

## Funding

This work was supported by the Clinician Scientist program of Else Kröner Research College ‘Phosphoproteome dynamics’ at Technische Universitaet Dresden (to BD), a grant from Deutsche Forschungsgemeinschaft (to RW, WI 5006/2-1), the MeDDrive program of the Faculty of Medicine Carl Gustav Carus at Technische Universitaet Dresden (to A-CF), the Saxon State Ministry for Science and the Arts (to ST), and the Dr. Robert Pfleger Foundation Bamberg (to SB and ST). The Facility ‘Molecular Analysis - Mass Spectrometry’ at the CMCB was generously supported by grants of the European Regional Development Fund (ERDF/EFRE) (Contract #100232736), the German Federal Ministry of Education and Research (BMBF, 03Z22EB1) and the German Research Foundation (DFG, INST 269/731-1 FUGG).

## Conflict of Interest

The authors declare that the research was conducted in the absence of any commercial or financial relationships that could be construed as a potential conflict of interest.

## Supplementary Material

Supplementary Figure 1 Immunofluorescent images of A375 cells. Panels show single channel images of A375 nuclear staining with DAPI, p75NTR/isotype staining, membrane staining with wheat germ agglutinin (WGA)-Alexa Fluor 594, and a merged channel image (**▪** nuclear staining with DAPI, **▪** p75NTR staining, **▪** membrane staining with WGA-AF594). **(A)** Staining with D4B3, **(B)** staining with MLR2, **(C)** staining with ME20.4. Scale bar = 50µm.

Supplementary Figure 2 Immunofluorescent images of permeabilized A375 cells. Panels show single channel images of A375 nuclear staining with DAPI, p75NTR/isotype staining, membrane staining with wheat germ agglutinin (WGA)-Alexa Fluor 594, and a merged channel image (**▪** nuclear staining with DAPI, **▪** p75NTR staining, **▪** membrane staining with WGA-AF594). Staining with D4B3, staining with MLR2, staining with ME20.4. Scale bar = 50µm.

Supplementary Figure 3 Immunofluorescent images of PMDC05 cells. Panels show single channel images of PMDC05 nuclear staining with DAPI, p75NTR/isotype staining, membrane staining with wheat germ agglutinin (WGA)-Alexa Fluor 594, and a merged channel image (**▪** nuclear staining with DAPI, **▪** p75NTR staining, **▪** membrane staining with WGA-AF594). **(A)** Staining with D4B3, **(B)** staining with MLR2, **(C)** staining with ME20.4. Scale bar = 50µm.

Supplementary Figure 4 Immunofluorescent images of permeabilized PMDC05 cells. Panels show single channel images of PMDC05 nuclear staining with DAPI, p75NTR/isotype staining, membrane staining with wheat germ agglutinin (WGA)-Alexa Fluor 594, and a merged channel image (**▪** nuclear staining with DAPI, **▪** p75NTR staining, **▪** membrane staining with WGA-AF594). Staining with D4B3, staining with MLR2, staining with ME20.4. Scale bar = 50µm.

Supplementary Figure 5 p75NTR expression in different cell lines. **(A)** FACS analysis of *wildtype* NIH/3T3, 293T and p75NTR overexpressing (OE) NIH/3T3 and 293T. In WT-NIH/3T3, no fluorescence shift is observed, while WT-293T show a minimal expression indicated by the slight shift in fluorescence. In OE cells, GFP fluorescence indicates a uniform transduction, and D4B3 stainings show a bimodal distribution. **▪** isotype control, **▪** p75NTR staining **(B)** *left:* D4B3 stainings of A375 and PMDC05 (**▪** isotype control, **▪** p75NTR staining); *right:* western blots comparing p75NTR expression in 293T-WT, 293T-OE, A375, B16 (murine melanoma), PMDC05 (numbers above the lanes correspond to the number of cells lysates were prepared from); detection antibody: D4B3, exposure time is indicated below the blots.

Supplementary Figure 6 p75NTR IP with reduced PMDC05 input. Western blots of acid (E1) and sample buffer (col) elution fractions from IP trials with ME20.4 and isotype matched control (CTL), and lysate. Numbers above fraction labels indicate the number of cells used as IP input. At approx. 70 kDa (red box), specific signals for p75NTR are detected with as little as 10^6^ cells. 15% of acid eluate (E1), 30% of sample buffer eluate (col) and lysate of 10^6^ cells were loaded onto the gel. *Detection:* primary antibody D4B3, secondary antibody anti-rabbit IgG-HRP (CST).

Supplementary Table 1 Ordering information of applied reagents and chemicals.

Supplementary Table 2 Ordering information of applied TaqMan assay probes.
