# Supplementary figures and images for: Listening to the Whispers in Neuroimmune Crosstalk: A Comprehensive Workflow to Investigate Neurotrophin Receptor p75NTR Under Endogenous, Low Abundance Conditions
Source: Front Immunol. 2021 Apr 16;12:648283. doi: 10.3389/fimmu.2021.648283 (PMC8085361; doi:10.3389/fimmu.2021.648283)

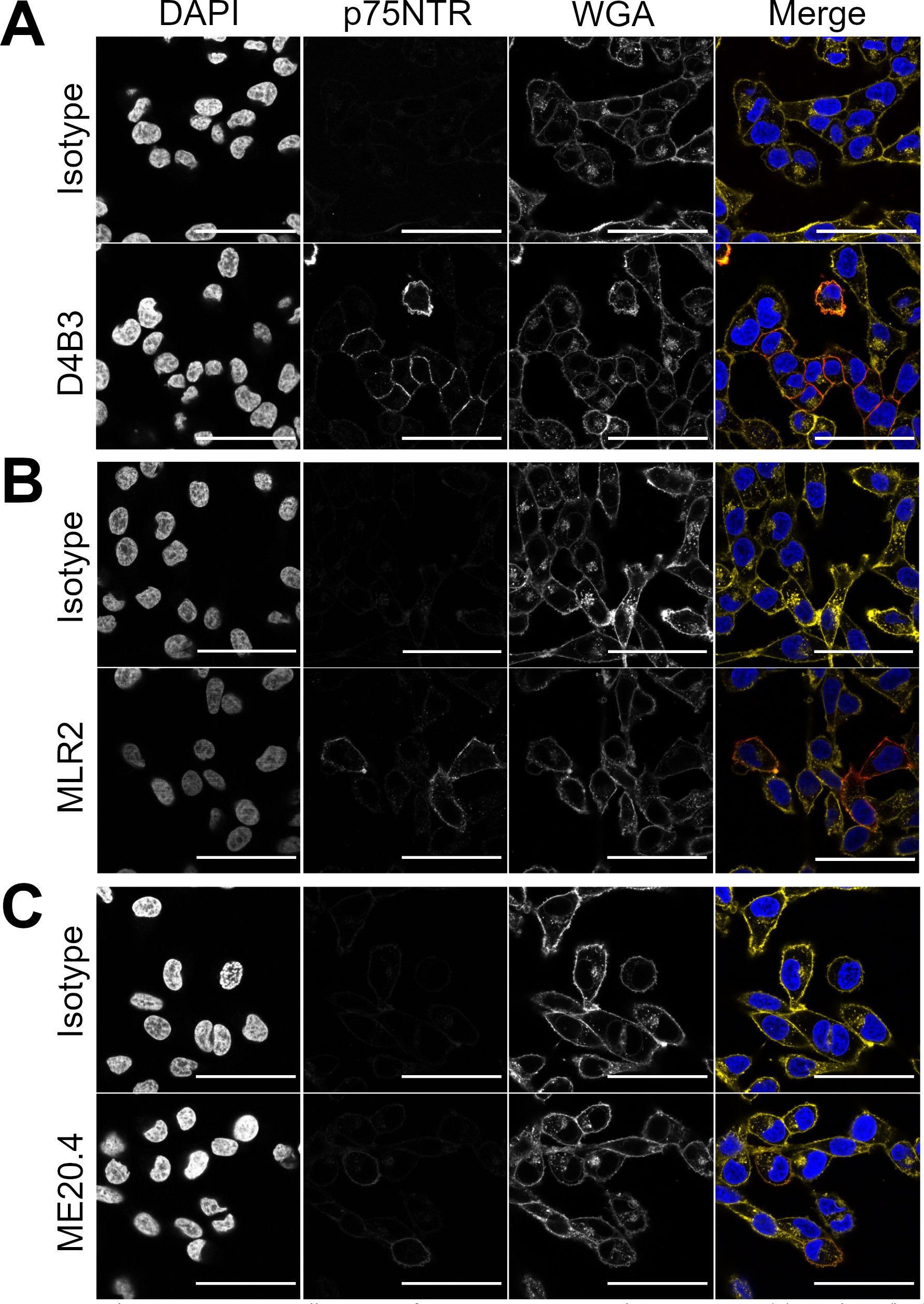

Supplement: Supplementary Figure 1 — Immunofluorescent images of A375 cells. Panels show single channel images of A375 nuclear staining with DAPI, p75NTR/isotype staining, membrane staining with wheat germ agglutinin (WGA)-Alexa Fluor 594, and a merged channel image (▪ nuclear staining with DAPI, ▪ p75NTR staining, ▪ membrane staining with WGA-AF594). (A) Staining with D4B3, (B) staining with MLR2, (C) staining with ME20.4. Scale bar = 50µm. [file Image_1.jpeg]

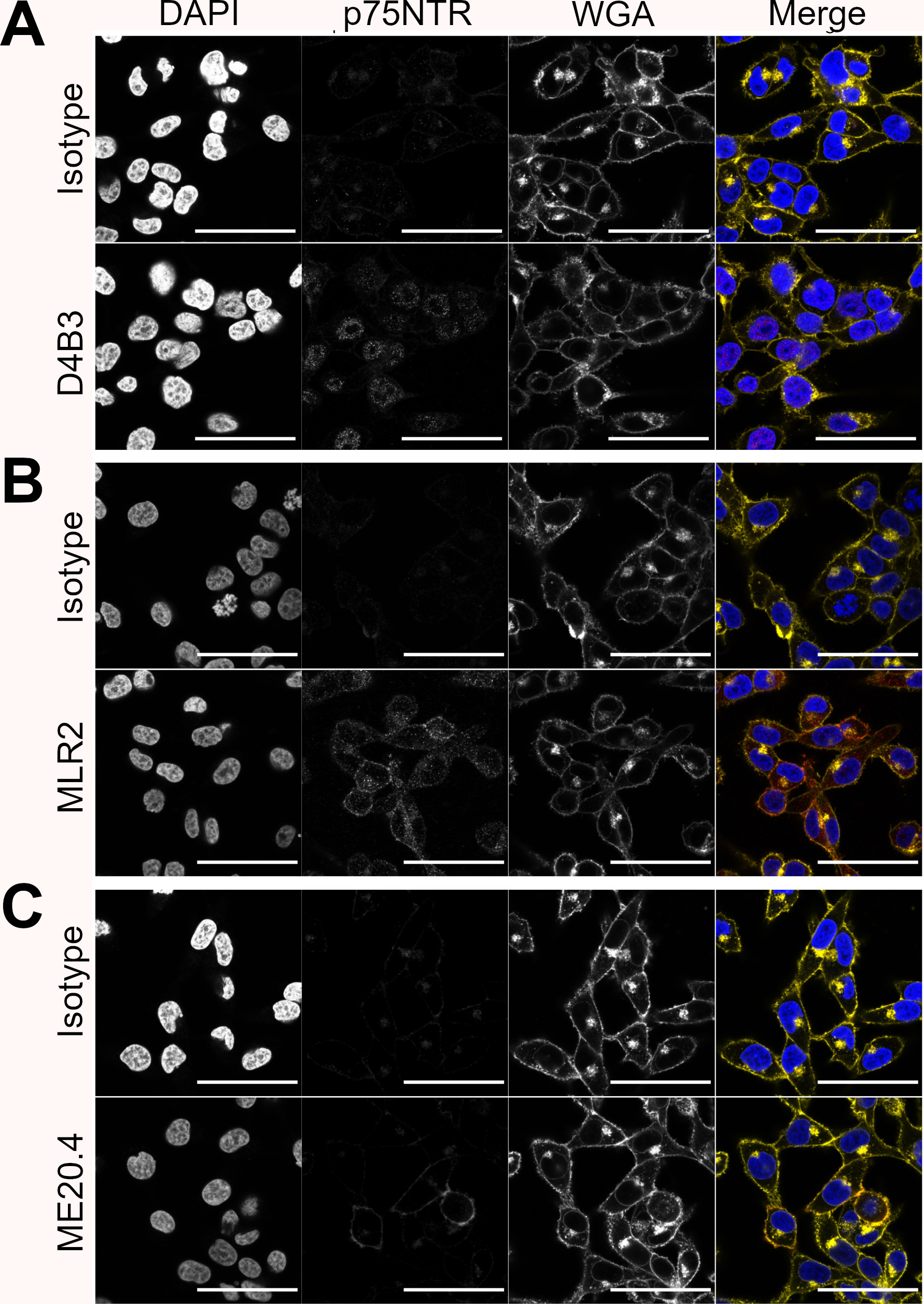

Supplement: Supplementary Figure 2 — Immunofluorescent images of permeabilized A375 cells. Panels show single channel images of A375 nuclear staining with DAPI, p75NTR/isotype staining, membrane staining with wheat germ agglutinin (WGA)-Alexa Fluor 594, and a merged channel image (▪ nuclear staining with DAPI, ▪ p75NTR staining, ▪ membrane staining with WGA-AF594). Staining with D4B3, staining with MLR2, staining with ME20.4. Scale bar = 50µm. [file Image_2.jpeg]

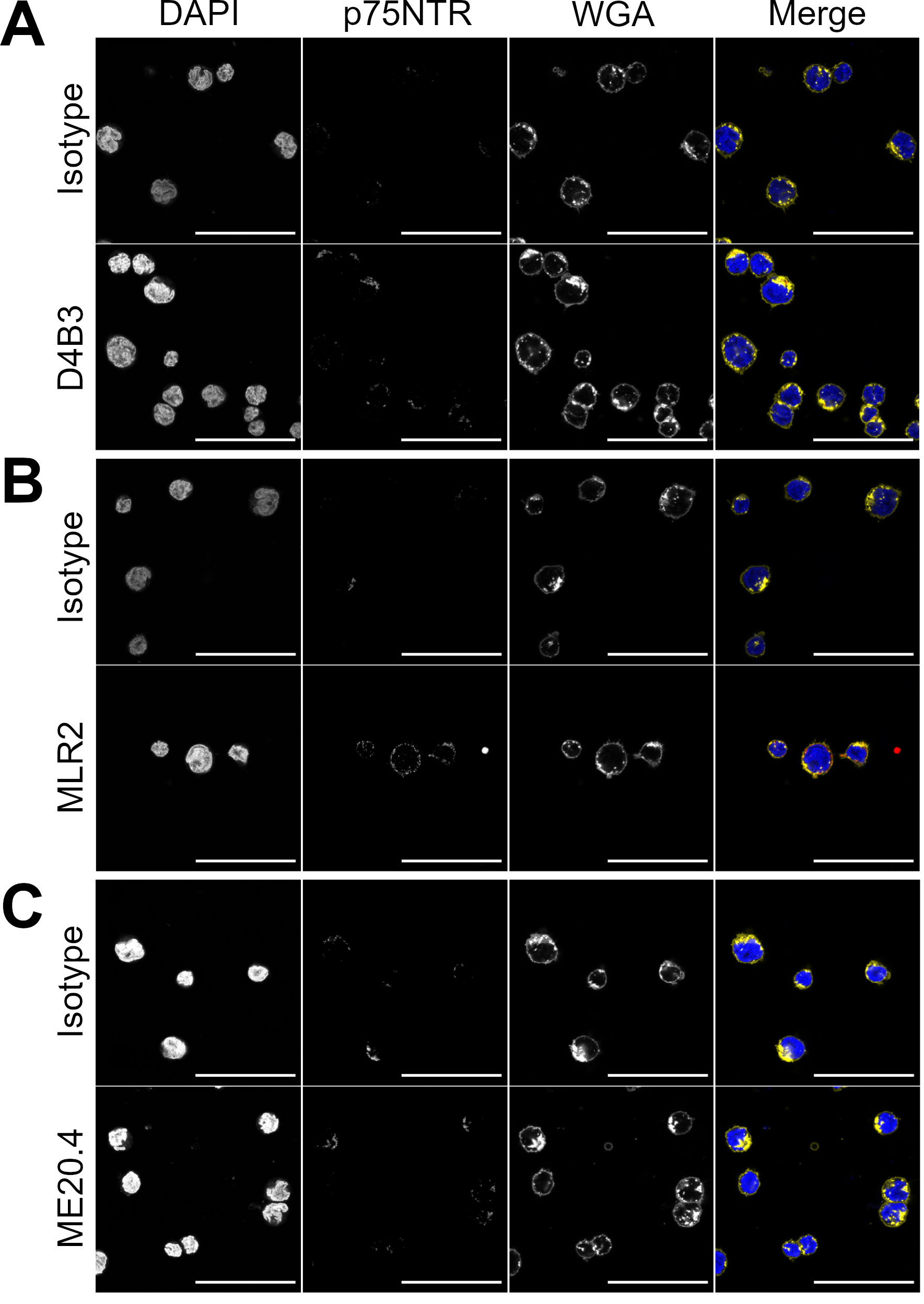

Supplement: Supplementary Figure 3 — Immunofluorescent images of PMDC05 cells. Panels show single channel images of PMDC05 nuclear staining with DAPI, p75NTR/isotype staining, membrane staining with wheat germ agglutinin (WGA)-Alexa Fluor 594, and a merged channel image (▪ nuclear staining with DAPI, ▪ p75NTR staining, ▪ membrane staining with WGA-AF594). (A) Staining with D4B3, (B) staining with MLR2, (C) staining with ME20.4. Scale bar = 50µm. [file Image_3.jpeg]

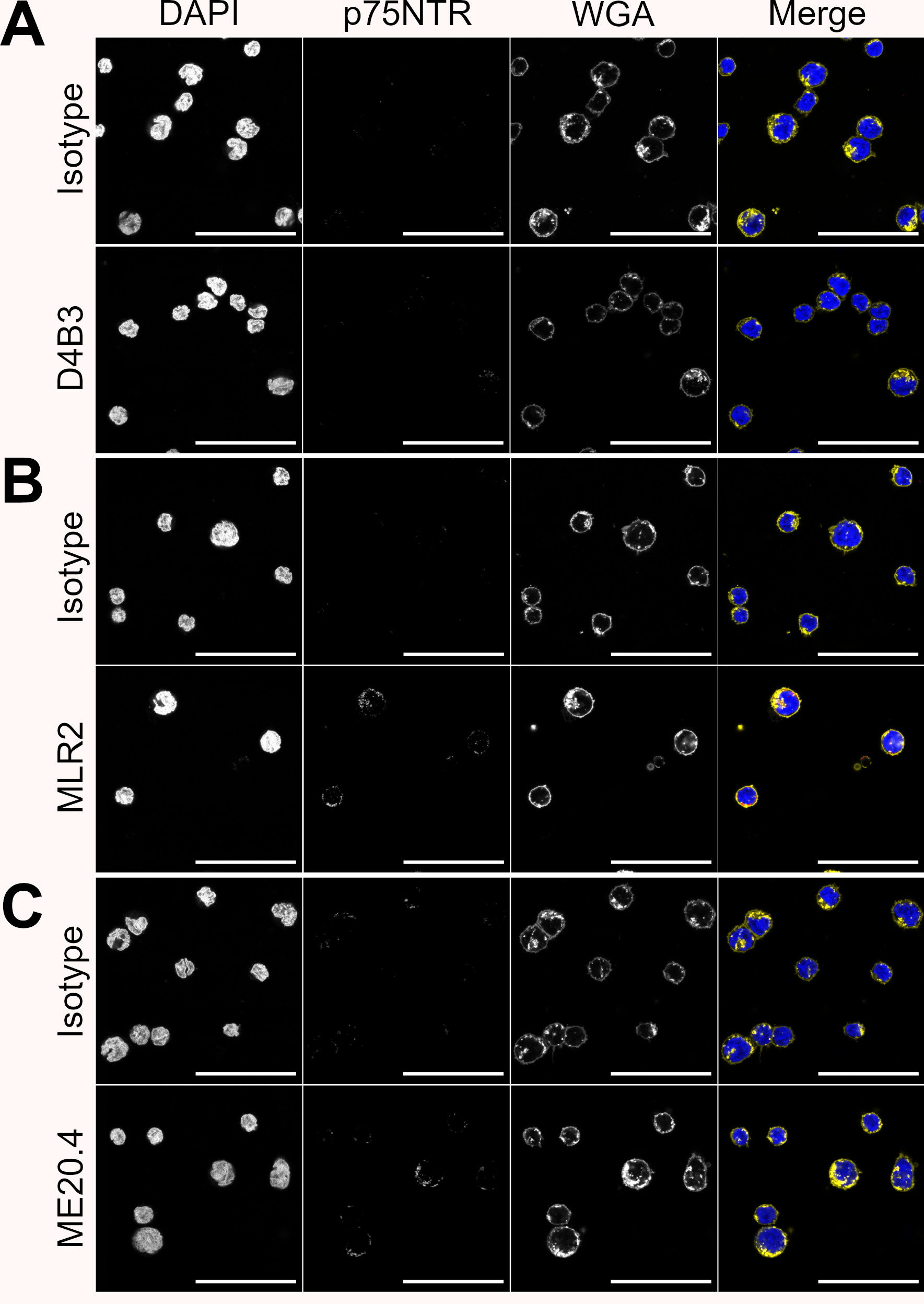

Supplement: Supplementary Figure 4 — Immunofluorescent images of permeabilized PMDC05 cells. Panels show single channel images of PMDC05 nuclear staining with DAPI, p75NTR/isotype staining, membrane staining with wheat germ agglutinin (WGA)-Alexa Fluor 594, and a merged channel image (▪ nuclear staining with DAPI, ▪ p75NTR staining, ▪ membrane staining with WGA-AF594). Staining with D4B3, staining with MLR2, staining with ME20.4. Scale bar = 50µm. [file Image_4.jpeg]

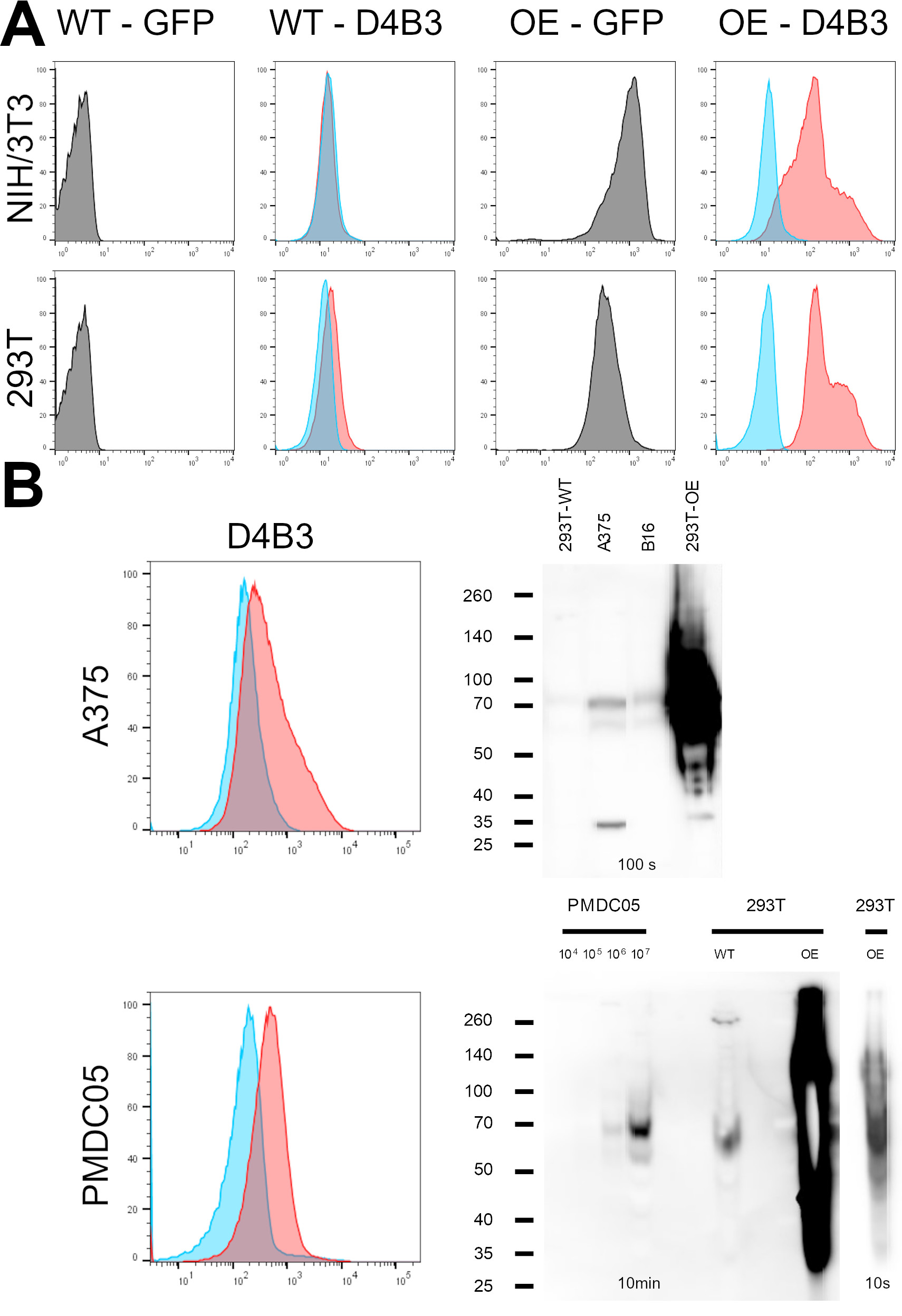

Supplement: Supplementary Figure 5 — p75NTR expression in different cell lines. (A) FACS analysis of wildtype NIH/3T3, 293T and p75NTR overexpressing (OE) NIH/3T3 and 293T. In WT-NIH/3T3, no fluorescence shift is observed, while WT-293T show a minimal expression indicated by the slight shift in fluorescence. In OE cells, GFP fluorescence indicates a uniform transduction, and D4B3 stainings show a bimodal distribution. ▪ isotype control, ▪ p75NTR staining (B) left: D4B3 stainings of A375 and PMDC05 (▪ isotype control, ▪ p75NTR staining); right: western blots comparing p75NTR expression in 293T-WT, 293T-OE, A375, B16 (murine melanoma), PMDC05 (numbers above the lanes correspond to the number of cells lysates were prepared from); detection antibody: D4B3, exposure time is indicated below the blots. [file Image_5.jpeg]

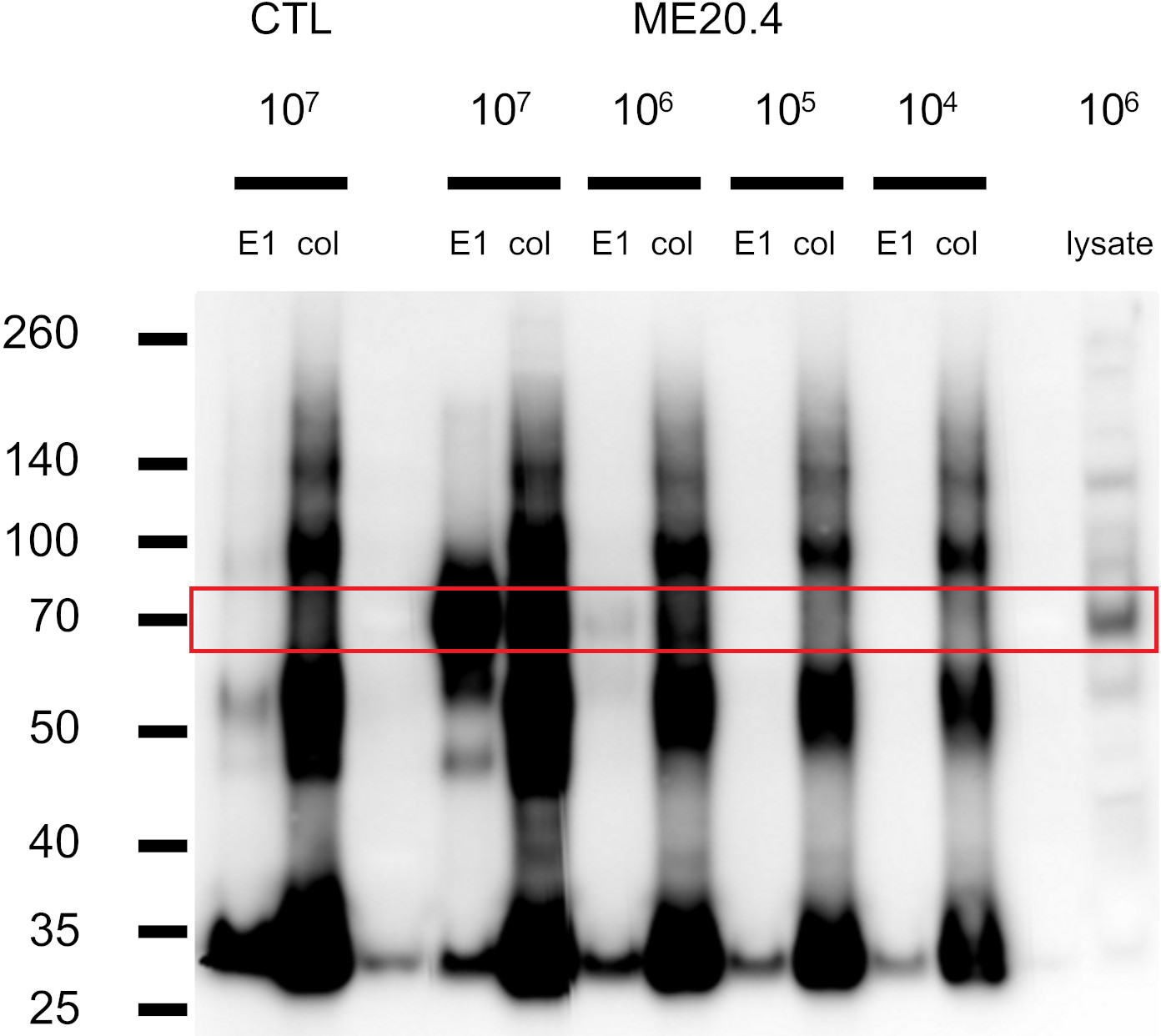

Supplement: Supplementary Figure 6 — p75NTR IP with reduced PMDC05 input. Western blots of acid (E1) and sample buffer (col) elution fractions from IP trials with ME20.4 and isotype matched control (CTL), and lysate. Numbers above fraction labels indicate the number of cells used as IP input. At approx. 70 kDa (red box), specific signals for p75NTR are detected with as little as 106 cells. 15% of acid eluate (E1), 30% of sample buffer eluate (col) and lysate of 106 cells were loaded onto the gel. Detection: primary antibody D4B3, secondary antibody anti-rabbit IgG-HRP (CST). [file Image_6.jpeg]
