# Supplementary material for: Listening to the Whispers in Neuroimmune Crosstalk: A Comprehensive Workflow to Investigate Neurotrophin Receptor p75NTR Under Endogenous, Low Abundance Conditions
Source: Front Immunol. 2021 Apr 16;12:648283. doi: 10.3389/fimmu.2021.648283 (PMC8085361; doi:10.3389/fimmu.2021.648283)
Supplement: Supplementary file 9 [file DataSheet_1.docx]

**Supplementary Information**

**Supplementary Methods – Generation of cell lines with transgenic p75NTR expression**

**Cell lines**

- Mouse embryonic fibroblast cell line NIH/3T3 was purchased from ATCC (CRL-1658).
- Human embryonic kidney cell line 293T was purchased from DSMZ-German Collection of Microorganisms and Cell Cultures (ACC 635).

**Recipes for media**

- DMEM complete: DMEM supplemented with 10% FCS, 4 mM GlutaMAX, 1 mM sodium pyruvate and 100 units mL^-1^ Penicillin-Streptomycin.
- IMDM complete: IMDM supplemented with 10% FCS, 4 mM GlutaMAX and 100 units mL^-1^ Penicillin-Streptomycin.

**Culture of human embryonic kidney cell line 293T**

1. Cultivate at a density of 4 × 10^5^ cells cm^-2^ in pre-warmed IMDM complete (0.2 mL cm^-2^) in an incubator (37°C, 95% humidity, 5% CO_2_).
2. Passaging (usually after 2-3 days):
3. Remove cell culture supernatant, gently add PBS (0.1 mL cm^-2^), swivel cell culture vessel and discard supernatant.
4. Add pre-warmed 21 µM trypsin solution (0.03 mL cm^-2^) and incubate at 37°C for 5 min, check cell detachment with microscope.
5. Add 3 volumes of IMDM complete.
6. Transfer cell suspension to a centrifuge tube and centrifuge at 300 × g for 8 min at room temperature.
7. Resuspend cells in pre-warmed IMDM complete and count cells (e.g. in a Neubauer chamber).
8. Adjust cells to desired concentration.

**Culture of murine embryonic fibroblast cell line NIH/3T3**

1. Cultivate at a density of 0.5 × 10^4^ cells cm^-2^ in pre-warmed DMEM complete (0.2 mL cm^-2^) in an incubator (37°C, 95% humidity, 5% CO_2_).
2. Passaging (usually after 2-3 days):
3. Remove cell culture supernatant, gently add PBS (0.1 mL cm^-2^), swivel cell culture vessel and discard supernatant.
4. Add pre-warmed 21 µM trypsin solution (0.03 mL cm^-2^) and incubate at 37°C for 5 min, check cell detachment with microscope.
5. Add 3 volumes of DMEM complete.
6. Transfer cell suspension to a centrifuge tube and centrifuge at 300 × g for 8 min at room temperature.
7. Resuspend cells in pre-warmed DMEM complete and count cells (e.g. in a Neubauer chamber).
8. Adjust cells to desired concentration.

**Production of lentiviral vector particles facilitating expression of human p75NTR coding sequence**

1. Prepare cell suspension of 293T cells (0.5 × 10^6^ cells mL^-1^) as described at section ‘Culture of human embryonic kidney cell line 293T’ in pre-warmed IMDM complete.
2. Cultivate 293T cells (0.2mL cell suspension cm^-2^ culture vessel surface) for around 16 hours in an incubator (37°C, 95% humidity, 5% CO_2_).

*Critical parameters:*

- Cell density of 75 to 80% at end of culture is critical for a high efficacy of virus vector particle production. Depending on visual control incubation period can be extended or shortened by 2 to 3 hours.

1. Preparation of plasmid mixture
   1. Calculate total volume of plasmid mixture as follows: 10µL mixture per cm^-2^ 293T culture vessel surface.
   2. For 100 µL plasmid mixture add lentiviral packaging plasmid psPAX2, pseudotyping vector pMD2.G (coding for VSV-G envelope) and lentiviral transfer vector pReceiver-Lv203_hsNGFR C-Flag-SV40-eGFP-IRES-puromycin at a ratio of 9:5:10 DNA copy numbers.
   3. Fill to final volume with IMDM without additives.
   4. Mix by vortexing.

*Critical parameters:*

- Molar ratio between applied plasmid components can be adapted to improve lentiviral vector particle production.
- Purity and concentration of applied plasmid preparation significantly affect transfection efficacy.
- Pseudotyping vector can be exchanged to modify tropism of prepared vector particles.

1. Preparation of PEI mixture
   1. Prepare a pre-dilution of 5mg PEI mL^-1^ ddH_2_O.
   2. Calculate total volume of PEI mixture as follows: 10µL mixture per cm^-2^ 293T culture vessel surface.
   3. Add 90.3µL of IMDM without additives to empty reaction tube.
   4. Add 5.7µL of PEI (5mg mL^-1^) solution to IMDM without touching reaction tube walls.
   5. Mix by vortexing.
2. Prepare DNA-PEI solution by mixing equal volumes (100 µL) of prepared plasmid and PEI mixtures in a reaction tube and incubate for 30 min at room temperature.
3. Add pre-warmed IMDM complete medium to DNA-PEI solution resulting in a total volume of 90µL per cm^-2^ 293T culture vessel surface. Mix by vortexing.
4. Replace culture media from 293T cells with prepared DNA-PEI-IMDM solution and incubate for around 16 hours in an incubator (37°C, 95% humidity, 5% CO_2_).

*Critical parameters:*

- Add DNA-PEI-IMDM solution over the top of the cell culture vessel avoid disturbance of 293T cell layer.
- Incubation time of 16 hours has proven to be optimal. However, shortening to 6 hours delivers usable amounts of vector particles.

1. Remove cell culture supernatant and replace with new pre-warmed IMDM complete medium (90µL per cm^-2^ 293T culture vessel surface) and incubate for around 16 hours in an incubator (37°C, 95% humidity, 5% CO_2_).
2. Harvest cell culture supernatant containing produced lentiviral vector particles.
3. Centrifuge (300g, 8', RT) harvested supernatant, aliquot and store at -80°C.

*Critical parameters:*

- This step is critical to remove remaining 293T cells from the harvested supernatant.
- Lentiviral vector particles can be stored for at least 1 year without loss of infectivity.
- To increase transduction rate, lentiviral vector particles can be concentrated by ultracentrifugation.

**Transduction of 293T and NIH/3T3 cells with lentiviral vector particles for overexpression of human p75NTR coding sequence**

1. Prepare a protamine sulfate solution (5mg mL^-1^ ddH2O).
2. Prepare an overnight culture of NIH/3T3 and 293T cells as described in respective sections.
3. Thaw prepared lentiviral vector particle and mix with equal volume of complete media (DMEM for NIH/3T3 or IMDM for 293T). Final volume should be 0.2mL cm^-2^ target culture vessel area.
4. Add protamine sulfate solution at a ratio of 1:1,000 to the particle-media mix.
5. Remove culture media from target cell containing vessels.
6. Add particle-media-protamine mix to the cells and incubate for around 16 hours in an incubator (37°C, 95% humidity, 5% CO_2_).
7. Replace lentiviral particle containing media, add fresh cell specific media and incubate (37°C; 5 v/v% CO2; 95% humidity) overnight.

**Supplementary Results**

**Comparative interpretation of p75NTR expression**

Mouse fibroblast cell line NIH/3T3 exhibited no detectable p75NTR transcript expression (data not shown). Human embryonic kidney cell line 293T exhibited a low p75NTR transcript expression (data not shown). Both cell lines were transduced with lentiviral particles to overexpress p75NTR (OE). As both wildtype (WT) cell lines did not express GFP the detectable GFP expression is evidence of the successful transduction (Fig. S5A). NIH/3T3 cells (WT) also did not show any detectable binding of p75NTR antibody D4B3. Cell line 293T (WT) exhibited a slight increase in D4B3 mediated fluorescence. These findings were in agreement with the respective RNA expression analyses. Both OE cell lines displayed a strong binding of p75NTR antibody D4B3. Aside from a significant shift in fluorescence intensity, the nearly bimodal distributions suggest a subpopulation with facilitated antibody binding. GFP fluorescence shows a unimodal distribution which indicates a uniform transduction.

Our findings show that a slight population shift in D4B3 fluorescence intensity corresponds to a low p75NTR expression. The nearly bimodal fluorescence intensity distributions could be interpreted as distinct subpopulations. Because the unimodal, narrow GFP fluorescence argues for a uniform transduction we speculate that the two “peaks” might represent different epitope accessibilities.

The left panels in Fig. S5B depict flow cytometry data of p75NTR expression in cell lines A375 and PMDC05. Both cell lines exhibited an increase in fluorescence intensity compared to isotype controls. The PMDC05 intensity distribution is similar to 293T (WT) data (Fig. S5A) whereas the pattern in A375 cells is reminiscent of the nearly bimodal distributions in 293T OE and NIH/3T3 OE. The right panels compare p75NTR detection by D4B3 in western blots of A375 and PMDC05 to 293T WT and 293T OE. The p75NTR expression in A375 and PMDC05 was slightly higher than in 293T WT. 293T OE displayed an intense antibody staining, even after short illumination times.

**IP experiments with decreasing lysate input**

The IP results with different decreasing lysate input (corresponding to 10^4^ to 10^7^ cells) are shown in Fig. S6. At approx. 70 kDa, a specific p75NTR band was detected. Two bright bands at approx. 55 and 30 kDa represent IgG heavy and light chains, respectively. Compared to Fig. S3, this illustrates the effects of different secondary antibodies in western blot detection.

**Supplementary figure legends**

**Supplementary figure S1: Immunofluorescent images of A375 cells.**

Panels show single channel images of A375 nuclear staining with DAPI, p75NTR/isotype staining, membrane staining with wheat germ agglutinin (WGA)-Alexa Fluor 594, and a merged channel image (**■** nuclear staining with DAPI, **■** p75NTR staining, **■** membrane staining with WGA-AF594). (A) Staining with D4B3, (B) staining with MLR2, (C) staining with ME20.4. Scale bar = 50µm.

**Supplementary figure S2: Immunofluorescent images of permeabilized A375 cells.**

Panels show single channel images of A375 nuclear staining with DAPI, p75NTR/isotype staining, membrane staining with wheat germ agglutinin (WGA)-Alexa Fluor 594, and a merged channel image (**■** nuclear staining with DAPI, **■** p75NTR staining, **■** membrane staining with WGA-AF594). Staining with D4B3, staining with MLR2, staining with ME20.4. Scale bar = 50µm.

**Supplementary figure S3: Immunofluorescent images of PMDC05 cells.**

Panels show single channel images of PMDC05 nuclear staining with DAPI, p75NTR/isotype staining, membrane staining with wheat germ agglutinin (WGA)-Alexa Fluor 594, and a merged channel image (**■** nuclear staining with DAPI, **■** p75NTR staining, **■** membrane staining with WGA-AF594). (A) Staining with D4B3, (B) staining with MLR2, (C) staining with ME20.4. Scale bar = 50µm.

**Supplementary figure S4: Immunofluorescent images of permeabilized PMDC05 cells.**

Panels show single channel images of PMDC05 nuclear staining with DAPI, p75NTR/isotype staining, membrane staining with wheat germ agglutinin (WGA)-Alexa Fluor 594, and a merged channel image (**■** nuclear staining with DAPI, **■** p75NTR staining, **■** membrane staining with WGA-AF594). Staining with D4B3, staining with MLR2, staining with ME20.4. Scale bar = 50µm.

**Supplementary figure S5: p75NTR expression in different cell lines.**

(A) FACS analysis of *wildtype* NIH/3T3, 293T and p75NTR overexpressing (OE) NIH/3T3 and 293T. In WT-NIH/3T3, no fluorescence shift is observed, while WT-293T show a minimal expression indicated by the slight shift in fluorescence. In OE cells, GFP fluorescence indicates a uniform transduction, and D4B3 stainings show a bimodal distribution. **■** isotype control, **■** p75NTR staining (B) *left:* D4B3 stainings of A375 and PMDC05 (**■** isotype control, **■** p75NTR staining); *right:* western blots comparing p75NTR expression in 293T-WT, 293T-OE, A375, B16 (murine melanoma), PMDC05 (numbers above the lanes correspond to the number of cells lysates were prepared from); detection antibody: D4B3, exposure time is indicated below the blots.

**Supplementary figure S6: p75NTR IP with reduced PMDC05 input.**

Western blots of acid (E1) and sample buffer (col) elution fractions from IP trials with ME20.4 and isotype matched control (CTL), and lysate. Numbers above fraction labels indicate the number of cells used as IP input. At approx. 70 kDa (red box), specific signals for p75NTR are detected with as little as 10^6^ cells. 15% of acid eluate (E1), 30% of sample buffer eluate (col) and lysate of 10^6^ cells were loaded onto the gel. *Detection:* primary antibody D4B3, secondary antibody anti-rabbit IgG-HRP (CST).

**Supplementary table S1: Ordering information of applied reagents and chemicals.**

**Supplementary table S2: Ordering information of applied TaqMan assay probes.**
